# Non-Epithelial Thymic Stromal Cells: Unsung Heroes in Thymus Organogenesis and T Cell Development

**DOI:** 10.3389/fimmu.2020.620894

**Published:** 2021-01-14

**Authors:** Takeshi Nitta, Hiroshi Takayanagi

**Affiliations:** Department of Immunology, Graduate School of Medicine and Faculty of Medicine, The University of Tokyo, Tokyo, Japan

**Keywords:** thymus, T cell, stromal cell, thymic epithelial cell, fibroblast, mesenchymal cell, repertoire selection

## Abstract

The stromal microenvironment in the thymus is essential for generating a functional T cell repertoire. Thymic epithelial cells (TECs) are numerically and phenotypically one of the most prominent stromal cell types in the thymus, and have been recognized as one of most unusual cell types in the body by virtue of their unique functions in the course of the positive and negative selection of developing T cells. In addition to TECs, there are other stromal cell types of mesenchymal origin, such as fibroblasts and endothelial cells. These mesenchymal stromal cells are not only components of the parenchymal and vascular architecture, but also have a pivotal role in controlling TEC development, although their functions have been less extensively explored than TECs. Here, we review both the historical studies on and recent advances in our understanding of the contribution of such non-TEC stromal cells to thymic organogenesis and T cell development. In particular, we highlight the recently discovered functional effect of thymic fibroblasts on T cell repertoire selection.

## Introduction

T cells, which are the central player in the acquired immune system, develop in the thymus (1). Supported by a three-dimensional framework composed of thymic stromal cells, immature T cells (called thymocytes) undergo proliferation, differentiation, and cell fate determination, and consequently give rise to mature T cells expressing a diverse T cell receptor (TCR) repertoire.

Thymus-seeding progenitor cells from the fetal liver or adult bone marrow enter into the thymus and give rise to Early T-cell progenitors (ETPs), followed by their differentiation from the CD4^−^CD8^−^ (double negative, DN) to the CD4^+^CD8^+^ (double positive, DP) stage and the expression of rearranged TCR on their cell surface (2). Interaction between the TCR and self-peptide/MHC complexes in the thymus dictates the fate of the developing thymocytes, namely, positive selection of CD4^+^CD8^−^ (CD4 single positive, CD4SP) or CD4^−^CD8^+^ (CD8 single positive, CD8SP) thymocytes, along with the negative selection of self-reactive cells or their diversion into regulatory T (Treg) cells (3). Such differentiation and selection events are controlled by the coordinated action of a set of thymic stromal cells that are localized in different areas of the thymus. TECs are one of the most prominent thymic stromal components (4, 5). The thymus is subdivided into two discrete regions, the cortex and medulla. The cortex is the outer region, where the cortical TECs (cTECs) provide a reticular meshwork that houses densely packed DN and DP thymocytes, while the medulla is the inner region with less densely localized SP thymocytes supported by medullary TECs (mTECs).

cTECs play key roles in the early events of T cell development, such as T cell lineage commitment, proliferation, migration, and the survival of immature thymocytes, by virtue of the production of the Notch ligand (DLL4), cytokines (SCF, IL-7), and chemokines (Cxcl12 and Ccl25) (6). Of particular importance in terms of T cell repertoire formation is that cTECs express a unique proteasome and lysosomal proteases, which enable the production and presentation of a unique set of self-peptides for the positive selection of a diverse TCR repertoire (7–11). In the subsequent steps, that are negative selection and Treg differentiation, mTECs play the dominant role. Functionally mature mTECs express a diverse set of genes that represent almost all of the coding transcripts, including those of the peripheral tissue-restricted antigens (TRAs) (12–14). This unique trait of mTECs ensures the negative selection and/or Treg conversion of self-reactive SP thymocytes that recognize such TRAs (15–20). mTECs also produce the chemokine CCL21 that induces the relocation of SP thymocytes from the cortex to the medulla, which promotes negative selection (21–24). Collectively, the thymic cortex promotes the generation of a diverse TCR repertoire, while the thymic medulla establishes the self-tolerance of T cells.

Several review articles in the current series “Thymic Epithelial Cells: New Insights in the Essential Driving Force of T Cell Differentiation”, as well as previous reviews, have provided detailed information on the function and heterogeneity of TECs (5). Although TECs are undoubtedly the key stromal component for controlling T cell development, numerous studies have also been accumulating that demonstrate the importance of thymic stromal cells other than TECs. In this review, we focus on such non-TEC thymic stromal cells in thymus organogenesis and T cell development.

## Overview of Thymic Stromal Cells

The thymus in mammals is made up of two lobes and is located in the upper anterior part of the chest between the lungs and on the heart. The outmost layer of the mouse thymus is covered with a capsule, which is composed of a monolayer of fibroblasts (capsular fibroblasts, capFbs). The cortical region directly under the capsule is called the subcapsular zone (SCZ), where proliferating DN thymocytes are localized (25). The SCZ contains a unique type of TECs (26, 27), but their function has been poorly elucidated. The cortex of the adult mouse thymus contains a network of cTECs (estimated as comprising ~10^6^ cells per mouse) that houses densely packed DP thymocytes (more than 10^8^ cells per mouse) (28). These cell number estimates are consistent with the observations that cTECs are very large cells with a three-dimensional reticular form so that a single cTEC adhere to hundreds of DP thymocytes. In particular, a fraction of cTECs form large multicellular complexes, termed ‘thymic nurse cells’, in that multiple DP thymocytes are enwrapped alive within intracellular vesicles of cTECs (29, 30). These unique cell-in-cell structures facilitate prolonged survival and continued TCRα rearrangement of enclosed DP thymocytes, likely contributing to the production of diverse TCR repertoire. Because of the difficulty in cell sorter-based isolation and single-cell-based analyses of such large cTECs, the functional heterogeneity of cTECs is still poorly understood and most likely underestimated (5). The cortex also contains dendritic cells that are sparsely distributed throughout the region and contribute to cortical negative selection (31).

Traveling inward from the cortex, there is a blood vessel-rich region called the cortico-medullary junction (CMJ), which is the site of the immigration of T-precursor cells and the emigration of mature SP thymocytes. The CMJ is also enriched with the lineage-committed progenitors of mTECs (termed junctional TECs) (32). In the medulla, mTECs and medullary fibroblasts (mFbs) form a reticular network that enmeshes SP thymocytes. The ratio of stromal cells to thymocytes is higher than that in the cortex. The number of mTECs is estimated to be ~2.5 × 10^6^ cells per mouse, which is still less than that of medullary SP thymocytes (estimated as ~1 × 10^7^ cells) but outnumbers the SP thymocytes newly generated in the adult mouse thymus per day (estimated to be ~1 × 10^6^), likely contributing to the efficient screening of SP thymocytes for self-reactivity (28). The medulla is also the place where dendritic cells and B cells are enriched, both of which contribute to the induction of self-tolerance (33–35).

The blood vasculature is also an important parenchymal component of the thymus that supplies oxygen and nutrients and ensures the import and export of cells. The vasculature in the thymus consists of morphologically and functionally distinct types of blood vessels. The cortex contains a network of capillaries, while the CMJ and medulla are enriched with arterioles and postcapillary venules (25, 36). A fraction of thymic endothelial cells are surrounded by pericytes, specialized contractile fibroblast-like cells expressing smooth muscle actin (α-SMA) (37).

In addition to the above types of stromal cells, histological studies have reported a variety of atypical cells that structurally resemble the epidermal epithelium, ciliated epithelium, neuroendocrine cells, muscle cells, or nerve cells in the thymus (38–43). Recent studies demonstrated that these cells can be categorized as subsets of differentiated mTECs that include keratinocyte-like mTECs forming Hassall’s corpuscles, thymic tuft cells, and neuroendocrine cell-like mTECs (44–47), suggesting the highly heterogeneous traits of mTECs and their possible contribution to the production of a diverse array of self-antigens for the establishment of self-tolerance. Also, certain neuron-associated genes such as *Nes*, *Pde1a*, and *Pde1b* are predominantly expressed in pericytes in the thymus [unpublished results based on transcriptome data (GEO: GSE147357)], although whether these cells and other nerve-like cells exert neural functions in the thymus is still unknown.

As animals age, the thymus undergoes a progressive atrophy called involution, mainly due to qualitative and quantitative degeneration of thymic stromal cells (4, 48). TEC is the thymic stromal cell type most affected by aging. In particular, mTECs are markedly compromised in cellularity and gene expression capability during aging (49). The age-dependent decline of TECs parallels with increased adipose tissue in the thymus. It was shown that TECs in aged mice can undergo epithelial-to-mesenchymal transition (EMT) to differentiate into fibroblasts and adipocytes (50, 51). How aging impacts mesenchymal thymic stromal cells and their role in thymic involution and adiposis awaits further studies.

To understand the developmental origin and function of these thymic stromal cells, it is necessary to trace them back to the organogenesis of the thymus.

## Thymic Organogenesis

**Figure 1:**
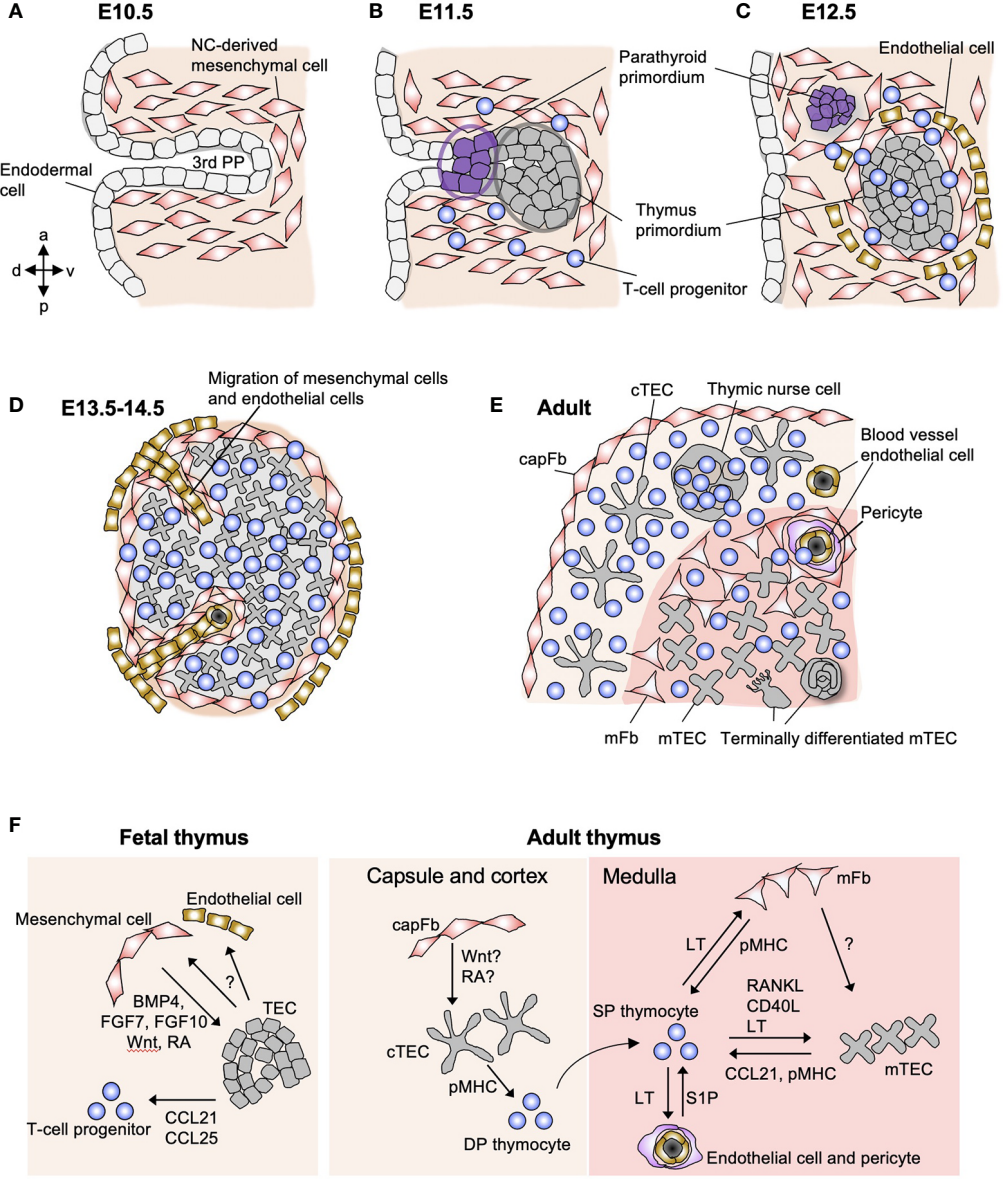
Schematic representation of stromal cells in the fetal and adult thymus. **(A)** The third pharyngeal pouch (PP) is a structure temporary formed in the E9.5–10.5 embryo, whose patterning is regulated by the surrounding NC-derived mesenchymal cells. **(B)** On E11.5, endoderm-derived epithelial cells form primordia of parathyroid gland and thymus. **(C)** Both primordia separate from the foregut on E12.5, being surrounded by NC-derived mesenchymal cells and mesoderm-derived endothelial cells. From E11.5 to E12.5, T-cell progenitors migrate into the thymic primordium by the coordinating action of chemokines, Ccl21 produced by parathyroid epithelium and Ccl25 produced by thymic epithelium. **(D)** On E13.5, endothelial cells and mesenchymal cells begin to migrate into the thymic primordium to form vascular network. **(E)** The intrathymic mesenchymal cells differentiate to reticular mFbs and blood vessel pericytes, while the perithymic mesenchymal cells remain outside of the epithelium and form the thymic capsule. **(F)** Interactions among stromal cells and developing T cells in fetal and adult thymus. In fetal thymus, NC-derived mesenchymal cells produce BMP4, FGF7, FGF10, Wnt ligands, and retinoic acid (RA) to promote the differentiation and proliferation of TECs. Fetal TECs induce inward migration of mesenchymal cells and endothelial cells via unknown mechanism. In adult thymus, mFbs control mTEC development via yet unidentified factors, suggesting that mFbs lie upstream in the hierarchy of stromal interaction in the medulla. The development of mature mFbs with unique gene expression is controlled by lymphotoxin (LT) signals provided by SP thymocytes. SP thymocytes also produce RANKL and CD40L as well as LT to induce differentiation and proliferation of mTECs. Both mFbs and mTECs contribute to the production of self-antigens for the induction of T cell tolerance. mTECs produce CCL21 to attract positively selected SP thymocytes from the cortex to the medulla. The emigration of mature T cells from the thymus is promoted by S1P produced by blood vessel pericytes.

The thymus develops from the third pharyngeal pouch (PP), which is formed by evagination of the endoderm-derived epithelial layer from the gut tube around embryonic day (E) 9.5–10.5 in C57BL/6 mice (52). In the third PP, the evaginated epithelial cells are surrounded by neural crest-derived mesenchymal cells (**Figure 1A**). These mesenchymal cells direct the patterning of the third PP through the production of soluble factors such as bone morphogenic protein-4 (BMP4) (53). The third PP is responsible for the origin of the parathyroid gland and the thymus (**Figure 1B**). The transcription factor FoxN1 starts to be expressed at E11.5 in the caudal ventral portion of the epithelial primordium and plays an essential role in thymus organogenesis, while Gcm2, another transcription factor expressed in the cranial dorsal portion, is required for the development of the parathyroid gland. Several other transcription factors expressed in mesenchymal cells (HoxA1, Eya1, Six1, Pax9, Tbx1, and Ripply3) in this region are also required for the patterning of the third PP and the subsequent development of the thymus. Genetic defects in these transcription factors results in thymus hypoplasia and severe immunodeficiency in humans (54).

On E12.5 in mice, the thymus primordium that is composed of epithelial and mesenchymal cells is detached from the pharynx (**Figure 1C**) and begins to migrate down the neck and into the mediastinum, where the two thymic lobes from the two sides fuse together in front of the trachea on E14.5. During these processes, the endodermal layer in the third PP differentiates into TECs. The mesenchymal cells surrounding the thymus primordium form the capsule that covers the surface of the adult thymus, while a fraction of these cells migrate into the epithelial rudiment in the period E12–14 to form the vascular and reticular architecture in the medulla of the adult thymus (**Figures 1D, E**). The thymic epithelial and mesenchymal components spatially and functionally interact in a coordinated manner to control thymus organogenesis and T cell development.

## The Fetal Thymic Mesenchyme Controls Thymic EPITHELIAL Cell Differentiation

An early study using a reaggregated organ culture technique demonstrated that fetal mesenchymal cells were required for the reaggregation of the thymic stroma and thymocytes and the development of DP thymocytes (55). In this experimental setting, the fetal mesenchymal cells can be replaced by fibroblasts treated so as to be metabolically inactive, suggesting that the fibroblast-associated extracellular matrix is the key element for supporting thymus organogenesis (56). The same group reported that when the mesenchymal layer was removed, the fetal thymic epithelium failed to undergo cellular expansion in both an *in vitro* organ culture (57) and *in vivo* organ transplantation (58). These findings suggest an essential role for thymic mesenchymal cells in controlling the capacity of T cell production.

The differentiation and expansion of fetal TECs are likely regulated by a combination of mesenchymal cell-derived signaling factors. BMP4, a soluble protein secreted by thymic mesenchyme and epithelium, is crucial for the development of both the thymus and parathyroid gland as well as TEC differentiation (53, 59). The fetal thymic mesenchyme also produces fibroblast growth factor-7 (FGF7), FGF10, and Wnt ligands that promote TEC differentiation and proliferation (58, 60–62). The expression of BMP4 and Wnt ligands in the fetal thymic mesenchyme is regulated by the transcription factor MafB (63). Thymic mesenchymal cells also serve as the major source of retinoic acid, which promotes the proliferation of cTECs (64).

Thymic mesenchymal cells diverge at an early stage (E13) to give rise to perithymic and intrathymic cell populations (57, 65) (**Figure 1D**). The perithymic cell population remains outside the organ and forms the thymic capsule, while the other population migrates into the thymus across the epithelial layers and differentiates into mFbs and pericytes (**Figure 1E**). Along with this migration, mesodermal progenitor cells enter into the thymic rudiment and differentiate into blood vessel endothelial cells in order to form the vascular network (66), and as a consequence, the vessels are connected to the peripheral vasculature at E15.5 (67), which switches the route of thymus ingress of T-cell progenitors from intraluminal crawling-dependent to bloodstream-dependent. The molecular mechanisms for generating the mesenchymal cell heterogeneity and the patterning of the vasculature remain unknown. It is also unclear which mesenchymal cell subset(s) are responsible for producing the key factors and controlling the fetal TECs. In the future, single-cell transcriptome analyses will be a powerful tool to decipher the heterogeneity of the fetal thymic mesenchyme in controlling thymus morphogenesis and TEC differentiation (68).

## Mesenchymal Stromal Cells in the Adult Thymus

In the adult thymus, neural crest-derived mesenchymal cells are found as fibroblastic cells predominantly localized in the capsule and medulla (69, 70) (**Figure 1E**). Some fibroblasts are sparsely distributed within the cortex. capFbs form a monolayer sheet that covers the surface of the thymic parenchyma that is filled with thymocytes and epithelial networks. In the medulla, neural crest-derived cells are subdivided into mFbs that form the reticular structure (71) and blood vessel adventitial layer (72) and pericytes that surround the endothelial cells.

Because of their abundance and unique structure in the thymus, thymic fibroblasts, similar to TECs, have been recognized as an important stromal component. Transcriptome analyses revealed that thymic fibroblasts express a unique set of genes for interactions with epithelial cells and are distinct from skin and bone fibroblasts, suggesting a pivotal role of these cells in the thymic microenvironment (73). Thymic fibroblasts also produce vasculogenic and angiogenic factors, consistent with their close localization and potential interaction with vascular endothelial cells (72). A series of studies have reported that thymic fibroblasts express a set of structural proteins and functional molecules, such as collagens, podoplanin/gp38, CD34, fibroblast-specific protein-1 (FSP1), platelet-derived growth factor receptor α (PDGFRα), PDGFRβ (58, 65, 71–76), and epitopes for the monoclonal antibodies MTS-15 and ER-TR7 (77, 78).

FSP1 (also known as S100a4) is reportedly not only a marker protein but also is responsible for the function of thymic fibroblast (74). Deletion of FSP1-expressing cells resulted in a prominent reduction in mTECs in both the steady state and regenerative phase after irradiation, suggesting that FSP1-expressing thymic fibroblasts are crucial for the maintenance and regeneration of mTECs. FSP1-deficient mice exhibited a smaller sized thymus and reduced number of mTECs compared with control mice. Furthermore, the addition of FSP1 protein increased the proliferation and maturation of mTECs in fetal thymus organ culture, indicating the role of FSP1 as a direct regulator of mTECs. The capacity of FSP1-expressing fibroblasts to control TECs might also be mediated by their ability to produce large amounts of FGF7. Collectively, this study demonstrates the pivotal role of FSP1-expressing fibroblasts in controlling mTECs.

However, the above mentioned thymic fibroblast-specific proteins or markers, including FSP1, cannot be used to distinguish two remotely localized fibroblast subsets, capFb and mFb. Whether thymic fibroblast subsets are functionally heterogeneous and how they make different contributions to TEC and T cell development remained open questions.

## Capsular Fibroblasts

Recently, we developed an enzymatic digestion method that allows the fractionation of thymic cells based on their intrathymic localization (79). Thymic fibroblasts were enriched in the fractions that correspond to the capsule and medulla, enabling the isolation of capFbs and mFbs. Among the differentially expressed genes, we identified *Dpp4*, a gene encoding the cell-surface protease dipeptidyl peptidase-4 (also called CD26), which is specifically expressed in capFbs and, consequently, established a means to separate capFbs (DPP4^+^ gp38^+^) and mFbs (DPP4^−^ gp38^+^) by flow cytometry and by histological analyses (79).

Both capFbs and mFbs highly express certain fibroblast-associated genes such as extracellular matrix proteins (*Col1a1*, *Col3a1*, *Col6a1*, *Dcn*, *Lum*, *Mgp*, and *Sparc*), extracellular proteases (*Htra1*, *Htra3*, *Mmp2*, *Mmp3*, *Mmp14*, and *Mmp23*), and protease inhibitors (*Serping1* and *Serpinh1*). These gene expression signatures are common to fibroblastic cell types in secondary lymphoid organs and are consistent with a previous report (73). capFbs but not mFbs highly express *Dpp4*, *Pi16*, *Sema3c*, *Sema3d*, and *Aldh1a2*. When compared with the transcriptome data of human thymic fibroblasts (46), it is suggested that the DPP4^+^ capFbs in the mouse thymus still contain (at least) two subpopulations: perilobular and interlobular cells. capFbs also express many Wnt family ligands and regulators (*Wnt2*, *Wnt5a*, *Wnt5b*, *Wnt9a*, *Wnt10b*, *Wnt11*, and *Sfrp2* and *Sfrp4*) at higher levels than those in mFbs and other thymic stromal cells, suggesting a role for capFbs in cTEC development via the regulation of Wnt signaling. The interplay between capFbs and subcapsular cTECs may be important for maintaining thymic architecture and thymopoietic niches, but uncovering its physiological significance and molecular basis still remains a challenge.

DPP4 is a useful marker for the detection and isolation of capFbs in the mouse thymus, although it has not yet been evaluated in the human thymus. It has been reported that DPP4 is expressed by activated fibroblasts in fibrotic tissues such as the skin of systemic sclerosis patients (80, 81). Genetic ablation and pharmacological inhibition of DPP4 ameliorated fibrosis in mice, indicating that DPP4 activity plays a role in fibroblast activation and tissue fibrosis. Whether the DPP4 is involved in the function of the thymic capsule remains to be elucidated.

## Medullary Fibroblasts

mFbs resemble fibroblastic reticular cells (FRCs) that form conduit-like network in secondary lymphoid organs, and they have also been known as thymic FRCs (71, 82). Transcriptome analyses on mFbs and LN FRCs revealed possible functional differences between these two morphologically similar cell types (79, 83). LN FRCs are subdivided into several types based on their expression of functional genes, including chemokines and cytokines: T-cell zone reticular cell (TRC) expressing *Ccl19*, *Ccl21a*, and *Il7*; follicular dendritic cell (FDC) expressing *Cxcl13*; marginal reticular cell (MRC) expressing *Tnfsf11* (RANKL); Cxcl12-expressing reticular cell (CRC); and medullary reticular cell (medRC) expressing *Cxcl12*, *Il6*, *Tnfsf13*, and *Tnfsf13b*. Most of these FRC-associated genes are not or just barely expressed in mFbs. In the thymus, RANKL is predominantly expressed in SP thymocytes to induce mTEC development (84), and Ccl19, Ccl21, Cxcl12, and IL-7 are produced by TECs so as to control the relocation and survival of thymocytes (85, 86). It is likely that the role played by FRCs in the LN is replaced by TECs in the thymus. mFbs highly express other sets of chemokines as well, such as Cx3cl1 and Cxcl14, that are barely expressed in LN FRCs or capFbs, possibly contributing to the regulation of cell migration in the thymic medulla. Thus, mFbs comprise a thymus-specific subset of fibroblasts that is functionally distinct from LN FRCs. It is possible that mFbs also consist of multiple subsets with different functions. A population of CD34^+^podoplanin^+^ mFbs are detected in adventitial layers that surround pericytes and endothelial cells, thus referred to as adventitial cells (72).

A set of genes that include certain collagens (*Col6a5*, *Col6a6*), matrix metalloprotease-9 (*Mmp9*), metabolic enzymes (*Hmgcs2*, *Ltc4s*, and *Qprt*), and TGFβ-binding proteins (*Ltbp1* and *Ltbp2*) are predominantly expressed in mFbs among the thymic stromal cell types. Notably, most of these mFb-associated genes are expressed under the control of the lymphotoxin signal. Lymphotoxin (LTα_1_β_2_), a TNF superfamily ligand, is primarily expressed by SP thymocytes in the thymus and binds to the lymphotoxin β receptor (LTβR) expressed in thymic stromal cells to induce intracellular signal transduction. Early studies reported that the LTβR expressed in thymic stroma is important for the induction of T cell tolerance, followed by later studies demonstrating the requirement of the LTβR in optimal mTEC differentiation (87–89). However, mice lacking LTβR specifically in TECs did not display any impact on T cell tolerance, indicating that the key target of lymphotoxin signaling in the context of tolerance induction should be non-TEC stromal cells (90, 91). We demonstrated that the LTβR expressed in mFbs is pivotal for the induction of T cell tolerance. LTβR-dependent, mFb-associated genes include certain TRAs, and mice specifically lacking LTβR in thymic fibroblasts exhibited a marked production of autoantibodies against those TRAs (79). The fibroblast-specific LTβR-deficient mice displayed signs of autoimmunity against peripheral tissues in a manner that was similar to systemic LTβR-deficient mice. These findings indicate that mFbs act as the primary source of certain self-antigens for the induction of T cell tolerance.

The number of self-antigens expressed in mFbs is less than that in mTECs, but mFbs express a set of cell type-specific antigens that mTECs cannot cover, contributing to the diversity of self-antigen expression so as to ensure the establishment of self-tolerance. Unlike mTECs, mFbs express MHC class I but not MHC class II or co-stimulatory molecules, suggesting that mFbs do not function as professional antigen-presenting cells. It is likely that mFb-derived self-antigens are transferred to and presented by thymic dendritic cells to induce T cell tolerance, just as a substantial portion of mTEC-derived antigens are indirectly presented by thymic dendritic cells (92, 93).

It is also possible that mFbs indirectly control T cell tolerance through the organization of mTEC development, since the fibroblast-specific deficiency of LTβR results in a reduction in the number of mTECs (79). Consistent with this, a previous report showed that the LTβR signal influences the localization of mFbs and their interaction with mTECs (75). In contrast, the loss of mTECs has no influence on mFb cellularity or gene expression (79). These findings indicate that mFbs are upstream in the hierarchy of stromal interactions in the medullary microenvironment. LTβR-dependent, mFb-associated genes such as extracellular matrixes or proteases might play various roles in organizing the cellularity and function of mTECs. LTβR signal also controls the expression of cell adhesion molecules ICAM-1 and VCAM-1 in mFbs, suggesting the role of mFbs in regulating immune cell trafficking in the thymus (72, 94).

## Cell–Cell Interactions in Thymic Medulla Formation

It is known that the formation of the thymic medulla is induced upon the development of SP thymocytes (33, 95). This lympho-stromal interplay is referred to as ‘thymic crosstalk’. The major mediator of the thymic crosstalk signal is RANKL, a TNF superfamily ligand expressed by positively selected SP thymocytes. Its receptor, RANK, is expressed predominantly on mTECs in the thymus. Mice lacking either RANKL or RANK exhibit a marked reduction of Aire^+^ mTECs and signs of autoimmunity, indicating the essential role of the RANKL-RANK axis in the induction of mTEC development and thereby T cell tolerance (84, 96, 97). RANKL is produced as a membrane-bound ligand and cleaved into the soluble form by proteases. As mice specifically lacking soluble RANKL have no phenotype in the thymic medulla, it is strongly suggested that membrane-bound RANKL is pivotal for mTEC differentiation (98). This is consistent with a previous finding that optimal differentiation of mTECs requires self-antigen-specific, cell-to-cell interactions with SP thymocytes (99).

The RANK signal activates the transcription factor NF-κB through signaling pathways mediated by TRAF6, NIK, and IκB-kinase (IKK) (100–104) and thereby induces mTEC differentiation and Aire expression in mTECs (105). RANKL-stimulated mTECs produce osteoprotegerin (OPG), an inhibitory decoy receptor for RANKL, to self-tune their differentiation and Aire expression (84, 106–108). SP thymocytes produce other TNFSF ligands, CD40L and lymphotoxin. CD40L acts cooperatively with RANKL to promote the generation of Aire^+^ mTECs (97), while lymphotoxin induces the differentiation of Aire^−^ mTEC subsets, including CCL21^+^ mTECs, Hassall’s corpuscles, and thymic tuft cells (109–113).

Furthermore, lymphotoxin is required for the development of the mature mFbs that express a set of mFb-associated genes. The lymphotoxin signal in mFbs also controls the cellularity of Aire^+^ mTECs (79). Along with the fact that SP thymocytes are the most abundant source of lymphotoxin as well as RANKL and CD40L in the thymus (84, 87), these series of findings provide a detailed picture of the thymic crosstalk in which developing SP thymocytes act on both mFbs and mTECs to establish the medullary microenvironment for selecting themselves (**Figure 1F**).

## The Thymic Vasculature and Blood–Thymus Barrier

After the vascularization takes place in the embryonic thymus, the endothelial cells that form the blood vessel network provide entry sites into the thymus for circulating ETPs. Thymic endothelial cells express a set of adhesion molecules, VCAM-1, ICAM-1, and P-selectin, that enable both the attachment of ETPs and their migration across the endothelium into the thymic microenvironment (114, 115).

A portion of the blood vessels in the CMJ and medullary regions are surrounded by extracellular matrix and pericytes to form perivascular spaces, through which mature T cells exit into the periphery. The T cell egress from the thymus is promoted by sphingosine-1-phosphate (S1P) and its receptor, S1P receptor 1 (S1P1). S1P is a lipid mediator that attracts mature SP thymocytes highly expressing S1P1 from the medullary region into the perivascular space. It has been shown that pericytes are responsible for the production of the S1P that promotes thymocyte egress (116). The trans-endothelial entry of ETPs and exit of mature T cells are regulated by the LTβR expressed in endothelial cells and pericytes (94, 117). Although the cellular source of LTβR ligands in the context of controlling cell traffic and the significance of this regulatory mechanism are not yet known, the LTβR may be a potential target to efficiently restore T cell production capacity in certain therapeutic situations such as bone marrow transplantation (117). Thymic endothelial cells also produce SCF/KitL to promote the survival of c-Kit-expressing ETPs, and this interaction is bi-directional in that membrane-bound SCF/KitL induces proliferation of thymic endothelial cells (118).

It has been established that the vascular permeability in the thymus is lower than that in other organs, suggesting the existence of a putative blood-thymus barrier (BTB) that limits the penetration of large circulating molecules such as proteins into the thymus (119, 120). Studies with intravenous injection of tracers demonstrated that the BTB is prominent in the cortex but weak in the medulla and there blood-borne molecules, including antigens, can enter the thymus (121). This mechanism may provide peripheral antigens entry into the medullary microenvironment so as to induce tolerance to these antigens, while in the cortex the blockade of peripheral antigens may be essential for the induction of positive selection by the cTEC-derived self-antigens. A recent report demonstrated that the role of the tight junction-forming protein claudin-5 (Cld5) in the BTB (122). Cld5 is expressed in capillaries at the cortex and arterioles at the CMJ, but not in postcapillary venules at the CMJ and medulla. Cld5^−^ postcapillary venule endothelial cells allow blood-borne molecules penetrate into the medullary parenchyma, while Cld5^+^ endothelial cells block their thymic influx. The Cld5^−^ endothelial cells are barely detectable in embryonic thymus but increase along postnatal development and aging, and serve as the site for the egress of mature T cells into the blood circulation in postnatal mice, suggesting that the expression profiles of Cld5^+^ in thymic endothelial cell subsets control the BTB and T cell gateway. Whether and how the BTB is regulated during infection to avoid the entry of infectious pathogens into the thymus is an important, but still open question (123). Blood-borne antigens and peripheral antigens are also transported to the thymus medulla by dendritic cells, contributing to the deletion of self-reactive T cell clones and the differentiation of Tregs (124–127).

## Conclusions

The studies on TEC development and TEC-specific characteristics conducted over the last two decades have remarkably advanced our understanding of T cell differentiation and repertoire selection in the thymus. On the other hand, non-TEC stromal cells, such as fibroblasts, endothelial cells, and pericytes, have been comparatively less investigated in view of T cell development and may be considered the ‘unsung heroes’ acting in the shadow of TECs. Based on the success of long-sought observations in histology and embryology, recent progress in and applications of single-cell transcriptome technologies have unveiled the functional diversity of such non-TEC stromal cells and highlighted their immunological functions. Along with pioneering studies on the regeneration of TECs (128–131), a better understanding of the cellular and molecular basis of the entire set of thymic stromal cells will provide valuable insights toward the *in vivo* reconstitution of the thymus for future therapeutic applications.

## Author Contributions

TN and HT wrote, drafted, and edited the manuscript. All authors contributed to the article and approved the submitted version.

## Funding

This research was supported by the Japan Society for Promotion of Science (JSPS) (KAKENHI 15H05703 to HT and 16H05202, 17H05788, 19H03485, and 19H04802 to TN), the CREST program of the Japan Agency for Medical Research and Development (AMED) (20gm1210008 to HT), and the Tokyo Society of Medical Science (to TN).

## Conflict of Interest

The authors declare that the research was conducted in the absence of any commercial or financial relationships that could be construed as a potential conflict of interest.
